# Association of Insurance Type With Inpatient Surgical 30-Day Readmissions, Emergency Department Visits/Observation Stays, and Costs

**DOI:** 10.1097/as9.0000000000000235

**Published:** 2023-02-14

**Authors:** Michael A. Jacobs, Jeongsoo Kim, Jasmine C. Tetley, Susanne Schmidt, Bradley B. Brimhall, Virginia Mika, Chen-Pin Wang, Laura S. Manuel, Paul Damien, Paula K. Shireman

**Affiliations:** From the * Department of Surgery, University of Texas Health San Antonio, San Antonio, TX; † Department of Population Health Sciences, University of Texas Health San Antonio, San Antonio, TX; ‡ Department of Pathology and Laboratory Medicine, University of Texas Health San Antonio, San Antonio, TX; § University Health, San Antonio, TX; ∥ Business Intelligence and Data Analytics, University of Texas Health Physicians, University of Texas Health San Antonio, San Antonio, TX; ¶ Department of Information, Risk, and Operations Management, Red McCombs School of Business, University of Texas, Austin, TX; # Departments of Primary Care & Rural Medicine and Medical Physiology, School of Medicine, Texas A&M Health, Bryan, TX.

**Keywords:** emergency department visits, healthcare disparities, observation stays, readmissions, social risk factors

## Abstract

**Objective:**

To assess the association of Private, Medicare (MC), and Medicaid/Uninsured (MU) insurance type with 30-day emergency department visits/observation stays (EDOS), readmissions, and costs in a safety-net hospital (SNH) serving diverse socioeconomic status patients.

**Background:**

MC’s hospital readmission reduction program (HRRP) disproportionately penalizes SNHs.

**Methods:**

This retrospective cohort study used inpatient National Surgical Quality Improvement Program (2013–2019) data merged with cost data. Frailty, expanded operative stress score, case status, and insurance type were used to predict odds of EDOS and readmissions, as well as index hospitalization costs.

**Results:**

The cohort had 1477 Private; 1164 MC; and 3488 MU cases with a patient mean age 52.1 years [SD = 14.7] and 46.8% of the cases were performed on male patients. MU [adjusted odds ratio (aOR) = 2.69, 95% confidence interval (CI) = 2.38–3.05, *P* < 0.001] and MC (aOR = 1.32, 95% CI = 1.11–1.56, *P* = 0.001) had increased odds of urgent/emergent surgeries and complications versus Private patients. Despite having similar frailty distributions, MU compared to Private patients had higher odds of EDOS (aOR = 1.71, 95% CI = 1.39–2.11, *P* < 0.001), and readmissions (aOR = 1.35, 95% CI = 1.11–1.65, *P* = 0.004), after adjusting for frailty, OSS, and case status, whereas MC patients had similar odds of EDOS and readmissions versus Private. Hospitalization variable cost %change was increased for MC (12.5%) and MU (5.9%), but MU was similar to Private after adjusting for urgent/emergent cases.

**Conclusions:**

Increased rates and odds of urgent/emergent cases in MU patients drive increased odds of complications and index hospitalization costs versus Private. SNHs care for higher cost populations while receiving lower reimbursements and are further penalized by the unintended consequences of HRRP. Increasing access to care, especially for MU patients, could reduce urgent/emergent surgeries resulting in fewer complications, EDOS/readmissions, and costs.

## Abstract 2

Mini-Abstract This inpatient cohort study used National Surgical Quality Improvement Program with cost data. Increased urgent/emergent case rates/odds in Medicaid/Uninsured patients drive increased complication odds and index hospitalization costs versus Private. Increasing access to care, especially for Medicaid/Uninsured patients, could reduce urgent/emergent surgeries resulting in fewer complications, emergency department visits/readmissions, and costs.

## INTRODUCTION

Medicare (MC) introduced the Hospital Readmission Reduction Program (HRRP) as a quality of care metric^1–3^ and to reduce healthcare expenditures.^4^ HRRP reduces MC payments for hospitals with higher than predicted 30-day readmissions by risk-adjusted models for select medical and surgical admissions.^4^ Reduced payments are applied to all fee-for-service MC diagnosis-related groups for the fiscal year. In Fiscal Year 2019, 75% of all hospitals were penalized.^3^ Although penalties are capped at up to 3%,^4^ this represents a significant burden on healthcare systems, especially for safety-net hospitals (SNHs) serving low-socioeconomic status (SES) populations.

HRRP succeeded in decreasing hospital readmissions.^5^ However, HRRP has fostered several unintended consequences with multiple publications questioning whether readmissions are an appropriate quality metric.^6–8^ First, pressure to reduce 30-day readmissions may incentivize hospitals to increase observation stays or discharges from emergency department (ED) visits, rather than having readmissions.^1,2^ These practices may have increased mortality for heart failure and pneumonia.^9^ Second, 30-day readmission risk can be attributed to patient factors^10^ outside of a provider’s control, such as frailty^11^ and social risk factors^12^ beyond dual MC-Medicaid eligibility.^3^ These factors are not included in HRRP risk adjustment models,^13–16^ penalizing hospitals serving more frail and/or socially disadvantaged patients. Frail and low-SES patients have higher rates of urgent or emergency surgeries,^17^ which are associated with worse outcomes.^18,19^ SNHs serve vulnerable populations^17^ and may provide the same quality of care,^20^ but are the most penalized by HRRP.^2,12,21^ Stratifying hospitals by dual MC-Medicaid eligibility reduces SNH penalties.^3^ However, 91.6% of low-SES/high-burden hospitals experienced penalties and the typical SNH still receives a 0.28% reduction in MC payments.^3^ Multiple studies classifying hospitals as having a high or low safety-net burden found that high-burden hospitals have higher readmission rates and higher costs,^17,22^ likely as a result of the population they serve.

However, the National Academy of Medicine states these factors need to be studied *within* healthcare systems serving a wide range of SES patients, rather than *across* hospitals with vastly different patient populations, to understand whether low-SES patients have worse outcomes due to lower quality of care or factors beyond provider control.^23,24^ Insurance status/type is a patient-level social risk factor, commonly used as a proxy measure of patient SES to predict surgical outcomes, with widely varying results.^25,26^ Variables such as insurance type,^25,26^ case status (elective, urgent, or emergent surgery),^18^ frailty,^19,27^ and increased surgical-induced physiologic stress^27^ have been associated with adverse outcomes across surgical specialties. Despite the developing literature on these topics, many studies^28–30^ are limited by their use of administrative claims data, which lack detailed data on patient risk factors and outcomes.^31–33^ Variations in assigning ICD-9/10 codes across institutions further adds to inaccuracies.^31^ Others use charge data^34^ or cost-to-charge ratios,^35^ not the actual, variable costs associated with patient healthcare use. Finally, prior studies focus on *either* readmissions^29,34^ or ED visits^30,35^ but do not include both factors.

We designed this study using high-quality, nurse-abstracted data for preoperative risk factors and complications from the National Surgical Quality Improvement Program (NSQIP)^31,32^ enriched with electronic health record (EHR)/billing data using *actual* variable costs to examine these associations including ED visits/observation stays (EDOS) and readmissions using the HRRP definition of 30 days from the date of discharge. We assessed the association of insurance type for inpatient surgical procedures with (1) actual variable costs of the index hospitalization, (2) 30-day EDOS, (3) 30-day readmissions, and (4) variable costs of EDOS and readmissions. Our cohort comes from one healthcare system serving patients over a wide range of SES, as recommended by the National Academy of Medicine to study outcomes within a healthcare system to understand the impact of social risk factors.^23,24^ We hypothesize that after risk adjustment for clinical and surgical factors, patients with Medicaid/Uninsured (UN) compared to Private insurance will be associated with increased 30-day EDOS, 30-day readmissions, and index hospitalization costs.

## MATERIALS AND METHODS

### Study Population and Data

This retrospective cohort study used data on all patients undergoing inpatient procedures present in the 2013-2019 American College of Surgeons NSQIP at an academic medical center and SNH following STROBE Reporting Guidelines. NSQIP registry provides standardized definitions of preoperative risk factors and complications^31^ and was used for cohort identification. The Institutional Review Board (IRB) of the University of Texas Health San Antonio approved this study and consent was waived.

### Estimating Patient Frailty/Premorbid Conditions

Frailty was measured using the recalibrated risk analysis index (RAI)^36^ using preoperative variables from NSQIP.^11,19^ RAI has been validated using NSQIP and Veterans Affairs Surgical Quality Improvement Program data sets.^11,36,37^ RAI exhibits collinearity^38^ with the Charlson Comorbidity Index. RAI is used as a single variable estimate of *patient*-level variability that overcomes barriers to model fit encountered by less parsimonious models.^19,27^ NSQIP stopped collecting cognitive decline variables after 2012. We treated this missing variable as not having any cognitive decline for all patients in the calculation of RAI, as previously reported.^36^ RAI scores were grouped into robust (≤20), normal (21–29), frail (30–39), and very frail (≥40) as previously described.^36^

### Expanded Operative Stress Score) Assignment, Case Status, and 30-Day Complications

The operative stress score (OSS) estimates surgical-induced physiologic stress of procedures across surgical specialties based on CPT codes by assigning a score ranging from 1 to 5 with 1 and 5 representing very low and very high physiological stress, respectively. We used the expanded OSS^19^ with 2343 CPT codes providing improved case coverage for nonmajority male populations compared to the original OSS.^27^ After excluding cases without an expanded OSS assigned to the principal CPT code, OSS was assigned using the highest score for all available procedures within each case.^19^ Case status was determined from NSQIP variables with urgent cases being defined as “no” responses to elective and emergency variables.^18^

Any complication was defined using the 20 NSQIP variables defining postoperative complications that occurred 30 days after the date of the index surgery (Supplemental Table 1, http://links.lww.com/AOSO/A201).

### 30-Day EDOS and Readmissions

NSQIP only tracks patients for 30 days after surgery and contains 30-day readmission variables from the date of surgery. We merged NSQIP with EHR data to determine readmissions and EDOS within 30 days of discharge from the index procedure’s hospitalization, to be consistent with the HRRP definition of 30-day readmissions.

### Insurance Type and Cost Data

The identified, local NSQIP data were merged with EHR and managerial accounting data to determine insurance type and cost of the index hospitalization, readmissions, and EDOS. Insurance type was categorized based upon billing data for the encounter supplemented by EHR data and defined as (1) Private insurance including Tricare and Workers Compensation; (2) MC; and (3) Vulnerable including dual enrollment in MC/Medicaid, Charity Care, self-pay, or county indigent care programs (Supplemental Table 2, http://links.lww.com/AOSO/A201). “Other” included encounters billed to the Veterans Administration, Department of Corrections, or self-pay with >1% of charges collected and were excluded (n = 87).

We defined variable costs as costs related directly to patient care occurring during the encounter, such as supplies and salaries, and included direct variable costs that vary directly with the quantity of resources provided for patient care. Variable costs were derived using direct measurements from a bottom-up approach, rather than calculated estimates derived from charges. Hospital fixed costs, outpatient, and professional fees were not included. We used variable costs, as fixed costs are not directly related to patient care and vary between hospitals.^39^ The natural logarithm of variable costs was used, as previously described,^40^ after adjusting costs to 2019 dollars using the Personal Health Care Index.^41^

### Exclusions

Cases were excluded due to (1) missing expanded OSS coverage of principal CPT code, (2) missing variables used to calculate the RAI other than cognitive decline, (3) other insurance status, and (4) missing or inaccurate cost variables (including readmissions external to the index hospital).

Patient mortality resulting in no or reduced chances of subsequent EDOS and readmissions were excluded, as previously described.^42^ Cases were excluded due to (1) death during the index hospitalization, (2) discharge to another acute care hospital, (3) discharge against medical advice, (4) death within 30 days of discharge when discharged to Hospice or Home on Hospice, and (5) death within 30 days of discharge without a 30-day EDOS or readmission. Two sensitivity analyses were performed adding exclusion groups 4 and 5 and 1 to 5 to the analysis to determine whether the association of insurance type was robust to cohort selection.

### Study Outcomes

Our clinical outcomes were the association of insurance type with (1) any complication, (2) EDOS, (3) readmissions, and (4) index hospitalization length of stay (LOS) on EDOS and readmission adjusted for frailty, OSS, and case status. Secondary analyses assessed the association of urgent/emergent cases with insurance type adjusted for frailty. Finally, the probabilities of EDOS and readmissions were calculated for lowest-risk versus highest-risk scenarios.

Our cost outcomes compared variable costs for the index surgery hospitalization using 3 discrete groups: (1) no 30-day EDOS/readmission, (2) 30-day readmission, and (3) 30-day EDOS without a 30-day readmission, patients with both a readmission and EDOS were assigned to the readmission group. We also assessed the variable costs of the first readmission and EDOS for each index case.

### Statistical Analysis

Categorical data was summarized using count and percentage, with continuous data using mean and standard deviation (SD). Chi-square tests and *F* tests were used to test for difference between groups for categorical and continuous variables. Kruskal-Wallis tests were used for the skewed LOS and variable costs for 1) index hospitalization, 2) EDOS and 3) readmissions. Logistic regression analyses were performed for case status and complications adjusting for a combination of RAI, OSS, case status, and insurance type. We calculated probabilities for lowest-risk versus highest-risk scenarios stratified by RAI/frailty from these final models. Natural logarithms were used to normalize the skewed LOS and variable costs, which reduces the impact of extreme values, as previously described.^40,43^ Percent change/relative difference was calculated using the exponential function; %change = (*e*^Estimate coefficients^ − 1) × 100. Analyses were performed using R version 4.1.0 (2021-05-18).

## RESULTS

### Population Demographics

Our cohort consisted of 6129 cases of inpatient procedures at a major urban SNH (Supplemental Fig. 1, http://links.lww.com/AOSO/A201). Most cases (Table 1) were performed on female (53.2%) and White patients (91.2%), with a majority identifying as Hispanic (67.4%). Cases were performed on MU insurance patients (56.9%), followed by Private (24.1%) and MC (19.0%). Most patients were robust (67.3%) and normal (23.7%) based on RAI scores. Only 7.5% and 1.5% of cases involved frail and very frail patients, respectively, with MC patients having higher rates of frailty.

**TABLE 1.** Patient Characteristics and Clinical Outcomes by Insurance Status

	Overall	Private	MC	MU	*P*
Number (%)*	6129	1477 (24.1)	1164 (19.0)	3488 (56.9)	
Sex (male)	2869 (46.8)	577 (39.1)	611 (52.5)	1681 (48.2)	**<0.001**
Age [mean (SD)]	52.14 (14.7)	48.27 (12.3)	66.56 (10.9)	48.96 (13.7)	**<0.001**
Race					**0.014**
White	5591 (91.2)	1339 (90.7)	1083 (93.0)	3169 (90.9)	
Black	386 (6.3)	87 (5.9)	59 (5.1)	240 (6.9)	
Other†	152 (2.5)	51 (3.5)	22 (1.9)	79 (2.3)	
Hispanic Ethnicity	4133 (67.4)	845 (57.2)	648 (55.7)	2640 (75.7)	**<0.001**
RAI					**<0.001**
Robust (≤20)	4123 (67.3)	1184 (80.2)	322 (27.7)	2617 (75.0)	
Normal (21–29)	1453 (23.7)	209 (14.2)	625 (53.7)	619 (17.7)	
Frail (30–39)	459 (7.5)	77 (5.2)	178 (15.3)	204 (5.8)	
Very frail (≥40)	94 (1.5)	7 (0.5)	39 (3.4)	48 (1.4)	
Case status					**<0.001**
Elective	2802 (45.7)	909 (61.5)	603 (51.8)	1290 (37.0)	
Urgent	2684 (43.8)	461 (31.2)	451 (38.7)	1772 (50.8)	
Emergent	643 (10.5)	107 (7.2)	110 (9.5)	426 (12.2)	
Expanded OSS (surgical-induced physiologic stress level)					**<0.001**
OSS1 (very low)	141 (2.3)	32 (2.2)	17 (1.5)	92 (2.6)	
OSS2 (low)	1476 (24.1)	274 (18.6)	242 (20.8)	960 (27.5)	
OSS3 (moderate)	3331 (54.3)	847 (57.3)	628 (54.0)	1856 (53.2)	
OSS4 (high)	1021 (16.7)	282 (19.1)	210 (18.0)	529 (15.2)	
OSS5 (very high)	160 (2.6)	42 (2.8)	67 (5.8)	51 (1.5)	
Any complication	1885 (30.8)	394 (26.7)	432 (37.1)	1059 (30.4)	**<0.001**
Length of stay (mean (SD))	8.25 (11.4)	6.38 (8.8)	9.37 (13.0)	8.67 (11.7)	**<0.001**
Median (Q1–Q3)	5 (3–9)	4 (2–7)	6 (3–11)	5 (3–10)	**<0.001** ‡
30-d EDOS	735 (12.0)	130 (8.8)	112 (9.6)	493 (14.1)	**<0.001**
30-d readmissions	892 (14.6)	167 (11.3)	185 (15.9)	540 (15.5)	**<0.001**

Readmissions and EDOS were evaluated independently; membership in one group does not exclude a case from membership in the other. Bolded *p*-values are significant at the <.05 level.

*Percent calculation by row, the rest of the percent calculations were by column.

†Other includes 10 cases missing race, 2 American Indian or Alaska Native, 78 Asian, 59 Multi-Racial, and 3 Native Hawaiian or Other Pacific Islander.

‡*P* value based on a Kruskal-Wallis test due to high skewedness of length of stay, all other *P* values calculated using chi-square tests and *F* tests.

Q1 indicates Quartile 1; Q3, Quartile 3.

### Increased Urgent/Emergent Cases and Complications in MC and MU patients

Urgent/emergency surgeries were higher in MU (63.0%) and MC (48.2%) patients compared to Private (38.4%; Table 1). Both MC [adjusted odds ratio (aOR) = 1.32, 95% confidence interval (CI) = 1.11–1.56, *P* = 0.001] and MU (aOR = 2.69, 95% CI = 2.38–3.05, *P* < 0.001) patients had higher odds of an urgent/emergent surgery compared to Private (Table 2). Complications were higher in MC (37.1%) compared to Private (26.7%) and MU (30.4%; Table 1). MC (aOR = 1.22, 95% CI = 1.01–1.46, *P* = 0.040) and MU (aOR = 1.24, 95% CI = 1.08–1.44, *P* = 0.003) patients also had higher odds of complications compared to Private (Table 3).

**TABLE 2.** Urgent/Emergent Case Status Adjusted for Frailty and Insurance Type

	Urgent/Emergent Cases		
Predictors	aOR	95% CI	*P*
RAI (Ref = normal 21–29)			
Robust (≤20)	0.82	0.72–0.93	**0.003**
Frail (30–39)	0.99	0.80–1.23	0.912
Very frail (≥40)	1.88	1.19–3.04	**0.008**
Insurance (Ref = private)			
MC	1.32	1.11–1.56	**0.001**
MU	2.69	2.38–3.05	**<0.001**

Ref indicates Reference value. Bolded *p*-values are significant at the <.05 level.

**TABLE 3.** Any Complication, 30-day EDOS, and 30-day Readmissions Adjusted for RAI, OSS, Case Status, Insurance Type and Any Complication

	Any Complication			30-d EDOS			30-d Readmissions		
	aOR	95% CI	*P*	aOR	95% CI	*P*	aOR	95% CI	*P*
RAI (Ref = normal 21–29)									
Robust (≤20)	0.64	0.56–0.74	**<0.001**	1.26	1.02–1.55	**0.030**	0.95	0.79–1.16	0.623
Frail (30-39)	1.33	1.06–1.65	**0.013**	1.04	0.74–1.45	0.823	1.23	0.93–1.63	0.147
Very Frail (≥40)	1.76	1.13–2.74	**0.012**	0.87	0.41–1.65	0.693	1.69	1.01–2.78	**0.041**
Expanded OSS									
OSS1–2^*^	0.61	0.53–0.71	**<0.001**	1.15	0.95–1.39	0.159	1.29	1.06–1.56	**0.012**
OSS4	2.87	2.48–3.33	**<0.001**	0.97	0.78–1.20	0.777	1.28	1.05–1.55	**0.014**
OSS5	4.02	2.88–5.66	**<0.001**	1.10	0.67–0.75	0.683	1.14	0.75–1.71	0.527
Urgent/emergent (Ref = Elective)	1.29	1.14–1.45	**<0.001**	0.90	0.76–1.06	0.210	1.02	0.86–1.20	0.852
Insurance (Ref = private)									
MC	1.22	1.01–1.46	**0.040**	1.15	0.86–1.52	0.344	1.13	0.88–1.46	0.339
MU	1.24	1.08–1.44	**0.003**	1.71	1.39–2.11	**<0.001**	1.35	1.11–1.65	**0.004**
Any complication				2.09	1.77–2.47	**<0.001**	7.03	5.97–8.29	**<0.001**

Readmissions and EDOS were evaluated independently; membership in one group does not exclude a case from membership in the other. Bolded *p*-values are significant at the <.05 level.

^*^OSS1 and OSS2 (very low and low stress surgeries) were combined due to small sample size of OSS1 procedures: OSS3 moderate stress, OSS4 high stress, and OSS5 very high stress.

Ref, reference value.

### Increased 30-day EDOS and Readmissions Among MU Patients

MU patients had the highest rates of EDOS (14.1%) and readmissions (15.5%; Table 1). MU patients also had higher odds of experiencing EDOS (aOR = 1.71, 95% CI = 1.39–2.11, *P* < 0.001) and readmissions (aOR = 1.35, 95% CI = 1.11–1.65, *P* = 0.004) compared to Private after adjusting for frailty, OSS, urgent/emergent cases, and complications (Table 3). MC patients had similar odds of EDOS and readmissions compared to Private. Complications increased the odds of EDOS (aOR = 2.09, 95% CI = 1.77–2.47, *P* < 0.001) and readmissions (aOR = 7.03, 95% CI = 5.97–8.29, *P* < 0.001).

### Lowest-Risk and Highest-Risk Probabilities for EDOS and Readmissions

The probabilities of EDOS and readmissions were estimated for the lowest-risk and highest-risk groups stratified by frailty (Table 4). As frailty increased, the difference between lowest and highest risk decreased for EDOS. For instance, robust patients at lowest-risk (Private, elective surgery, no complications) versus highest-risk (MU, urgent/emergent surgery, any complication) had an EDOS probability of 7.4% and 20.5%, respectively, a difference of 13.1%. In contrast, as frailty increased, the difference between lowest- and highest-risk increased for readmissions. Very frail patients at lowest risk versus highest risk had a readmission probability of 7.8% and 44.8%, respectively, a difference of 37.0%.

**TABLE 4.** Lowest-Risk Versus Highest-Risk Scenarios on Probability of EDOS and Readmissions for OSS3 (Moderate Stress Surgeries) Stratified by Frailty

	EDOS Probability Lowest-Risk	EDOS Probability Highest-Risk	Probability Differences Highest-Lowest Risk
	Private Insurance, Elective Surgery, No Complications (%)	Medicaid/Uninsured, Urgent/Emergent Surgery, Any Complication (%)	(%)
RAI			
Robust (≤20)	7.4	20.5	13.1
Normal (21–29)	6.0	17.0	11.0
Frail (30–39)	6.2	17.6	11.4
Very frail (≥40)	5.3	15.2	9.9
**Probability difference robust-very frail (%**)	2.1	5.3	
	Readmission Probability Lowest-Risk	Readmission Probability Highest-Risk	Probability Differences Highest-Lowest Risk
	Private insurance, Elective surgery,No complications (%)	Medicaid/Uninsured, Urgent/emergent surgery,Any Complication (%)	(%)`
RAI			
Robust (≤20)	4.5	31.3	26.8
Normal (21–29)	4.7	32.4	27.6
Frail (30–39)	5.8	37.1	31.3
Very Frail (≥40)	7.8	44.8	37.0
**Probability difference robust-very frail (%**)	−3.3	−13.5	

Models for EDOS and Readmissions from Table 3 used to estimate probabilities using the most common (54.3%) moderate stress surgery group (OSS3).

### Increased Index Hospitalization Variable Costs among Patients with a 30-Day EDOS or Readmission

The %change of variable costs was decreased for robust compared to normal patients (Table 5; M1–M3). The %change was lower for OSS1–2 and higher for OSS4 and OSS5 compared to OSS3 cases. MC (14.8%) and MU patients (7.8%) had higher %changes in variable costs compared to Private (Table 5; M1), but MU patients had similar %change to Private after adjusting for urgent/emergent cases (Table 5; M3). Patients with a readmissions or EDOS had a 37.2% and 12.8% higher %change, respectively, than those without either an EDOS or readmission as the reference group (Table 5; M1). However, after adjusting for complications, EDOS and readmission groups had similar costs to the reference group (Table 5; M2).

**TABLE 5.** Index Hospitalization Variable Costs Using 3 Nested Models (M1–M3) Adjusted for RAI, OSS, Insurance, 30-Day Readmissions, 30-Day EDOS, Any Complication, and Case Status

	log(Index Variable Costs) M1				log(Index Variable Costs) M2				log(Index Variable Costs) M3			
	%	Est	95% CI	*P*	%	Est	95% CI	*P*	%	Est	95% CI	*P*
Intercept		9.11	9.05 to 9.17	**<0.001**		8.95	8.90 to 9.00	**<0.001**		8.87	8.81 to 8.92	**<0.001**
RAI (Ref = Normal 21–29)												
Robust	−25.33	−0.29	−0.34 to −0.24	**<0.001**	−21.08	−0.24	−0.28 to −0.19	**<0.001**	−20.10	−0.22	−0.27 to −0.18	**<0.001**
Frail	3.22	0.03	−0.04 to 0.11	0.416	0.18	0.00	−0.07 to 0.07	0.959	−0.17	-0.00	−0.07 to 0.07	0.962
VF	12.39	0.12	−0.03 to 0.27	0.131	5.86	0.06	−0.08 to 0.20	0.426	1.93	0.02	−0.12 to 0.16	0.787
Expanded OSS (Ref = OSS3)												
OSS1–2^*^	−21.15	−0.24	−0.28 to −0.19	**<0.001**	−16.88	−0.18	−0.22 to −0.14	**<0.001**	−21.82	−0.25	−0.29 to −0.21	**<0.001**
OSS4	48.85	0.40	0.35 to 0.45	**<0.001**	30.33	0.26	0.22 to 0.31	**<0.001**	32.96	0.28	0.24 to 0.33	**<0.001**
OSS5	64.92	0.50	0.38 to 0.68	**<0.001**	38.08	0.32	0.21 to 0.43	**<0.001**	46.72	0.38	0.28 to 0.49	**<0.001**
Insurance (Ref = private)												
MC	14.79	0.14	0.08 to 0.20	**<0.001**	12.52	0.12	0.06 to 0.17	**<0.001**	11.15	0.11	0.05 to 0.16	**<0.001**
MU	7.79	0.07	0.03 to 0.12	**<0.001**	5.91	0.06	0.02 to 0.10	**0.006**	1.38	0.01	−0.03 to 0.06	0.515
30-d Readmissions and EDOS (Ref = no readmission or EDOS)^†^												
Readm	37.20	0.32	0.26 to 0.37	**<0.001**	2.37	0.02	−0.03 to 0.07	0.374	2.38	0.02	−0.03 to 0.07	0.366
EDOS	12.76	0.12	0.06 to 0.18	**<0.001**	1.71	0.02	−0.04 to 0.07	0.566	2.08	0.02	−0.04 to 0.08	0.481
Any complication					96.30	0.67	0.63 to 0.71	**<0.001**	93.99	0.66	0.62 to 0.70	**<0.001**
Urgent/emergent (Ref = elective)									23.77	0.21	0.18 to 0.25	**<0.001**

% change is calculated with marginal change of Log(variable costs) for one unit of each variable change below: (e ^(intercept+estimated coefficients)^ −  e^intercept^)/e^intercept^ × 100, which is equal to (e ^estimated coefficients^ − 1) × 100. Bolded *p*-values are significant at the <.05 level.

^*^OSS1 and OSS2 (very low and low stress surgeries) were combined due to small sample size of OSS1 procedures, OSS3 moderate stress, OSS4 high stress, and OSS5 very high stress.

^†^Patients with both a readmission and EDOS were assigned to the readmission group; readmissions (n = 892) and EDOS (n = 587).

% indicates % change; Est, estimates; Readm, readmissions; Ref, reference value; VF, very frail.

**FIGURE 1.**
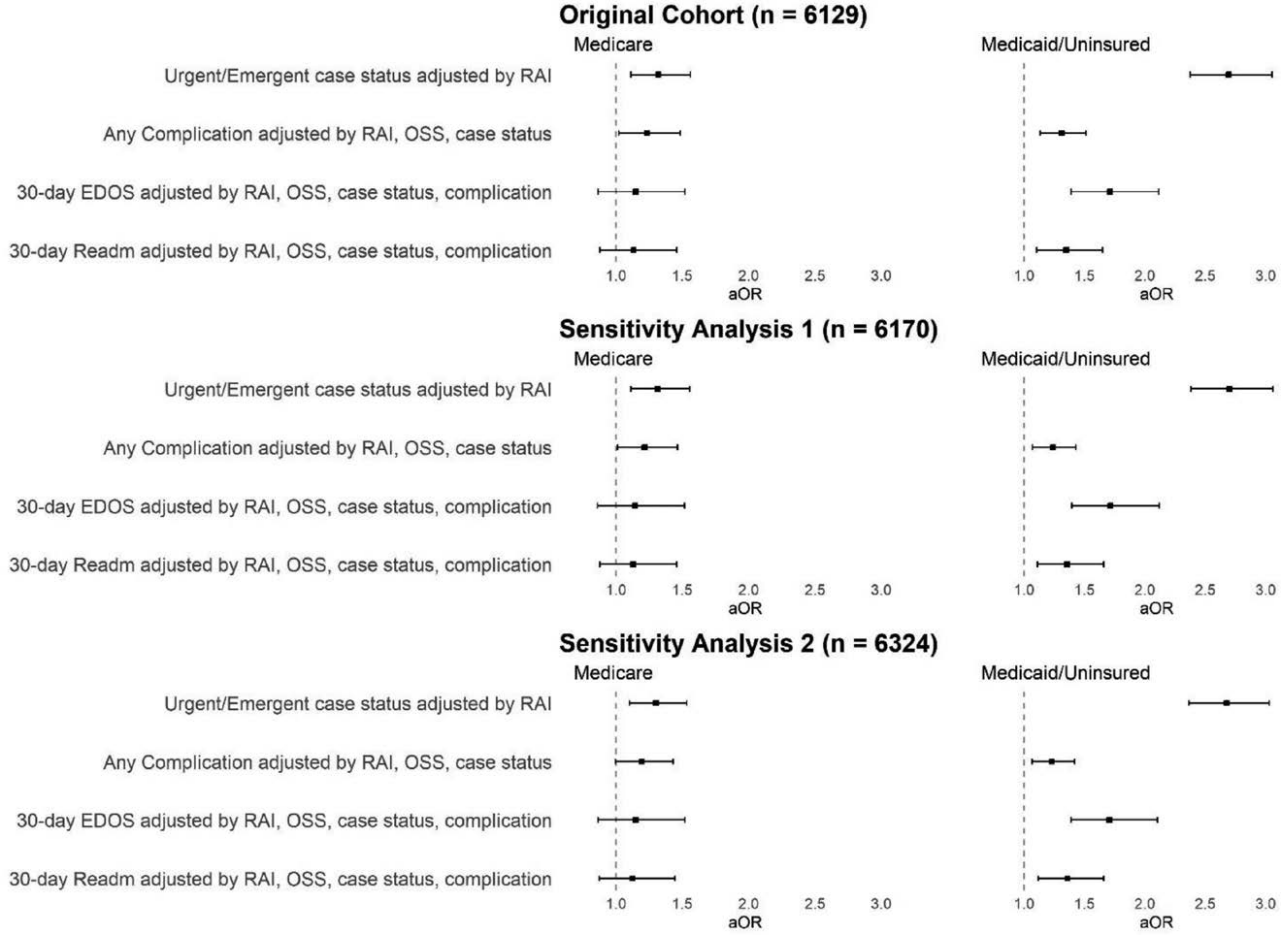
Cohort and sensitivity analyses of adjusted odds ratios for MC and MU insurance groups compared to Private for case status, any complication, 30-day EDOS and 30-day readmissions. aOR for MC and MU Insurance groups (reference Private group) for the study cohort and two sensitivity analyses. Cases were excluded from the cohort due to (1) death during the index hospitalization, (2) being discharged to another acute care hospital, (3) discharge against medical advice, (4) death within 30 days of discharge when discharged to Hospice or Home on Hospice, and (5) death within 30 days of discharge without a 30-day EDOS or readmission. Two sensitivity analyses were performed adding exclusion groups 4 and 5 and 1–5 to the analysis to determine whether the association of insurance type was robust to cohort selection. Readm indicates readmissions

Two sensitivity analyses increased the cohort to 6170 and 6324 cases showed similar results for insurance type for urgent/emergent case status, any complication, EDOS, readmissions (Fig. 1), and index hospitalization variable costs (Supplemental Fig. 2, http://links.lww.com/AOSO/A201) compared to our study cohort of 6129 cases.

### Increased Index Hospitalization LOS Among Patients With a 30-day EDOS or Readmission

The %change of LOS was decreased for robust compared to normal patients (Supplemental Table 3, M1–M3, http://links.lww.com/AOSO/A201). The %change was lower for OSS1–2 and higher for OSS4 and OSS5 compared to OSS3 cases. Patients with a readmission or EDOS had a 34.7% and 10.6% higher relative difference (Supplemental Table 3, M1, http://links.lww.com/AOSO/A201), respectively, than those without either a 30-day EDOS or readmission. After adjusting for complications, these groups had similar index LOS (Supplemental Table 3, M2, http://links.lww.com/AOSO/A201). MC (12.1%) and MU patients (14.2%) had higher %changes in LOS compared to Private even after adjusting for any complication (76.4%) and urgent/emergent cases (62.4%) (Supplemental Table 3, M3, http://links.lww.com/AOSO/A201).

### Similar EDOS and Readmission Costs Between Private, MC, and MU patients

The %change of the first EDOS and first readmission variable costs was similar for MC and MU compared to Private (Supplemental Table 4, http://links.lww.com/AOSO/A201). The %change of the first EDOS or readmission variable costs increased for cases experiencing any complication by 33.5% and 50.3%, respectively, and was decreased for robust compared to normal patients.

### Mean and Median Index Hospitalization Variable Costs by Insurance Type and OSS

Descriptive statistics for mean and median dollars for the index hospitalization were reported due to the right skewed variable costs. Variable costs increased with increasing OSS and were significantly different between insurance types overall and for all OSS groups except OSS5 (Table 6). Mean and median variable costs were higher for MC patients in OSS1–2 cases compared to the similar values for Private and MU. MC, MU and Private variable costs were highest, intermediate and lowest, respectively, for OSS3 cases. OSS4 cases were lowest for Private compared to similarly higher costs in MC and MU. Cases in MU patients had the highest cost with intermediate and lowest costs for MC and Private, respectively. Variable costs for elective (Supplemental Table 5, http://links.lww.com/AOSO/A201) were lower with less variation for all insurance types at all OSS groups compared to urgent/emergent (Supplemental Table 6, http://links.lww.com/AOSO/A201) cases.

**TABLE 6.** Mean and Median Index Hospitalization Variable Costs ($) for All Cases by Insurance Type Stratified by the Expanded Operative Stress Score

	Overall	Private	MC	MU	*P*
Number (%)^*^	6129	1477 (24.1)	1164 (19.0)	3488 (56.9)	
All cases costs Mean (SD)	12,545 (16,236)	11,241 (15,964)	14,998 (15,487)	12,279 (16,511)	**<0.001**
Q1	4974	4730	6298	4825	
Median	7841	7015	10,224	7568	
Q3	13,587	11,796	17,481	13,249	
Expanded OSS1–2^*^ Mean (SD)	9882 (12,975)	9034 (12,867)	12,630 (15,866)	9453 (12,114)	**<0.001**
Q1	3679	3362	4741	3672	
Median	5881	5172	8536	5709	
Q3	10,887	8,999	14,526	10,405	
Expanded OSS3 Mean (SD)	12,007 (16,335)	10,399 (14,399)	14,511 (16,001)	11,894 (17,164)	**<0.001**
Q1	4970	4705	5806	4946	
Median	7240	6320	8855	7231	
Q3	12,505	10,136	16,216	12,220	
Expanded OSS4 Mean (SD)	17,522 (19,200)	15,453 (22,215)	18,424 (14,196)	18,267 (19,145)	**<0.001**
Q1	8450	7699	9179	8528	
Median	11,879	10,503	12,875	12,022	
Q3	17,895	14,543	22,885	18,617	
Expanded OSS5 Mean (SD)	18,906 (14,758)	16,005 (9272)	17,984 (10,043)	22,504 (21,623)	0.220
Q1	11,576	11,020	11,357	12,304	
Median	14,505	13,777	15,516	14,438	
Q3	19,774	16,109	21,843	24,662	

Kruskal-Wallis test used for *P* values due to highly skewed cost data.Bolded *p*-values are significant at the <.05 level.

^*^OSS1 and OSS2 (very low and low stress surgeries) were combined due to small sample size of OSS1 procedures, OSS3 moderate stress, OSS4 high stress, and OSS5 very high stress.

Q1 indicates Quartile 1; Q3, Quartile 3.

## DISCUSSION

This study provides a comprehensive assessment of EDOS, readmissions, and variable costs across a single healthcare system including multiple surgical specialties/procedures serving a wide range of SES patients. Demographics of the Private and MU insurance type patients were similar regarding age and frailty scores, in contrast to MC patients who were older with higher frailty scores (Table 1). Increased rates and odds of urgent/emergent cases in MU (aOR = 2.69) and MC (aOR = 1.32) populations drive increased odds of complications, index hospitalization costs and LOS versus Private. However, despite similarly increased odds of complications in MC (aOR = 1.22) and MU (aOR = 1.24) patients, MU, but not MC patients, had higher 30-day readmission and EDOS rates and odds even after adjusting for frailty, operative stress, urgent/emergent cases and complications. Our results are similar to a prior study suggesting that SES is an independent risk factor for hospital readmissions.^10^ However, HRRP models do not adequately risk adjust for SES.^13,14^

The use of HRRP as a metric for low-quality care^1–3^ has been heavily criticized.^6–8^ Readmissions are largely driven by patient factors,^10^ urgent/emergency surgeries,^17^ and complications arising after discharge.^44^ SNHs have higher readmission rates^3^ and have also been shown to provide similar quality of care.^20^ Lower readmission rates may not indicate high-quality care as decreased readmission rates can be accompanied by increased EDOS.^28,29^ Failure to appropriately readmit patients can increase mortality,^1,2^ especially for patients originally admitted for heart failure and pneumonia.^9^ Readmissions in the current study were consistent with increased index hospitalization costs and LOS for patients undergoing coronary artery bypass graft surgery.^45^

Our study demonstrated the clinical significance of insurance type and case status. Robust patients at lowest-risk (Private, elective surgery, no complications) versus highest-risk (MU, urgent/emergent surgery, any complication) had an EDOS probability of 7.4% and 20.5%, respectively, a difference of 13.1%. In contrast, readmission probabilities were highest in very frail patients with a difference of 37.0% for lowest- versus highest-risk scenarios. We speculate that robust, low-SES patients are more likely to use the ED for routine/primary care,^30^ whereas older and frail patients are more likely to be readmitted secondary to their multiple medical comorbidities. SNH care for low-SES populations with Medicaid or no insurance. Our study shows the increased probabilities and increased costs of EDOS and readmissions borne by SNH.

Using readmissions as a care quality metric has serious consequences. Multiple studies demonstrate that SNH and academic medical centers receive disproportionate HRRP penalties^13,14,21^; further widening disparities between SNH, caring for vulnerable populations, and hospitals serving affluent communities.^21,46^ Poor communities exist under the burdens of inequalities in insurance coverage, education, and poverty while being treated at hospitals with less funding/resources,^14,21^ limiting their access to care. Our study shows that patients having a readmission or EDOS have higher index hospitalization variable costs which become similar after adjusting for complications. Additionally, increased rates and odds of urgent/emergent procedures in MU patients also drive increased complications, costs, and EDOS/readmissions. Thus, SNH operate under a triple burden; (1) higher index hospitalization costs, (2) increased readmissions and EDOS encounters leading to more costs, and (3) penalties for worse outcomes. Like SNH, academic medical centers also serve as healthcare providers for low-SES patients, and this trend continues to grow,^47^ making the healthcare system under study a particularly strong example of SES’s effect on surgical outcomes.

EDOS have similar associations as readmissions, with MU patients having increased index hospitalization costs and LOS. Unlike readmissions, care provided in many ED visits could be accomplished in lower-cost settings, suggesting that many ED visits are preventable.^48^ However, providers often advise their patients to go to the ED for surgical complications.^49^ Postoperative ED visits occurred in 17.3% in MC patients within 30 days after hospital discharge and 4.4% of patients had multiple ED visits.^50^ Low-SES patients often use the ED for routine care,^30^ creating yet another cost burden for SNH.

Consistent with prior studies, we observed variation in index hospitalization costs by insurance type^40^ with complications (94% change) having the highest associated costs.^22^ In this study, MU insurance patients had increased index hospitalization costs that were similar to Private after adjusting for urgent/emergent cases, whereas MC patients had 11.2% change even after adjusting for case status. Mean variable costs for urgent/emergent cases were substantially higher for all OSS groups versus elective cases. Insurance plans pay at different rates, and uninsured patients provide minimal, if any, revenue. MC and MU groups had increased urgent/emergent cases, complications, and costs, yet provide lower reimbursement than Private insurance.

### Limitations

This study is a retrospective review and did not establish causal relationships. NSQIP provides a random sample of a broad range of surgeries but does not include all procedures for a healthcare system. Encounters occurring at outside hospitals may be missing from our data set leading to misclassification of cases without an EDOS or readmission. Only the first readmission and EDOS costs were examined; MU patients might have increased readmission and EDOS encounters and costs if all 30-day encounters were included. The data are derived from one healthcare system which may restrict generalizability. However, as this study has no equivalent published in the literature, this study can affect the national debate on presentation acuity and access to care in public policy and risk adjustment.

## CONCLUSIONS

Increased rates and odds of urgent/emergent cases in MU (aOR = 2.69) and MC (aOR = 1.32) populations drive increased odds of complications, index hospitalization costs, and LOS versus Private insurance patients. However, despite similarly increased odds of complications in MC (aOR = 1.22) and MU (aOR = 1.24) patients, 30-day EDOS, and readmissions odds were only increased in MU compared to Private patients. SNH care for higher cost populations while receiving lower reimbursements and are further penalized by the unintended consequences of value-based programs such as HRRP. Increasing access to care, especially for MU patients, could reduce urgent/emergent surgeries resulting in fewer complications, EDOS, readmissions and costs.

## ACKNOWLEDGMENT

P.K.S., J.K., M.A.J., and L.S.M. had full access to the data and take responsibility for the integrity of the data and the accuracy of the data analysis. Concept and design: P.K.S., S.S., M.A.J., J.K., B.B.B., and P.D. Acquisition, analysis, or interpretation of data, Critical revision of the manuscript for important intellectual content, and Administrative, technical, or material support: All authors. Drafting of the manuscript: M.A.J. and P.K.S. Statistical analysis: M.A.J., J.K., C.-P.W., and P.D. Obtained funding: P.K.S., B.B.B., S.S., P.D., and C.-P.W. Supervision: P.K.S.

## Supplementary Material


