# Supplementary material for: Association of Insurance Type With Inpatient Surgical 30-Day Readmissions, Emergency Department Visits/Observation Stays, and Costs
Source: Ann Surg Open. 2023 Feb 14;4(1):e235. doi: 10.1097/as9.0000000000000235 (PMC10427129; doi:10.1097/as9.0000000000000235)
Supplement: Supplementary file 1 [file as9-4-e235-s001.pdf]

Supplemental Digital Content (SDC)

SDCTable1. National Surgical Quality Improvement Program (NSQIP) Variables Used for Any Complication

SDCTable2. Distribution of Medicaid/Uninsured Insurance Type

SDCFigure1. Flow Diagram of Study Cohort

SDCFigure2. Cohort and Sensitivity Analyses of Log-Linear Regression Coefficients for Medicare and Medicaid/Uninsured Insurance Groups Compared to Private for Index Hospitalization Variable Costs

SDCTable3. Index Hospitalization Length of Stay Adjusted for RAI, OSS, Insurance, 30-day Readmissions, 30-day EDOS, Any Complication and Case Status

SDCTable4. Variable Costs for 1st 30-day EDOS and 1st 30-day Readmission Adjusted for RAI, OSS, Case Status, Insurance and Any Complication

SDCTable5. Average and Median Index Hospitalization Variable Costs ($) for Elective Cases by Insurance Type Stratified by the Expanded Operative Stress Score

SDCTable6. Average and Median Index Hospitalization Variable Costs ($) for Urgent/Emergent Cases by Insurance Type Stratified by the Expanded Operative Stress Score

SDCTable1. National Surgical Quality Improvement Program (NSQIP) Variables Used for Any Complication

Variable Name	NSQIP Definition
REINTUB	Intubation intraoperatively or within 30 days after surgery
PULEMBOL	New diagnosis of a pulmonary embolism within 30 days after surgery
FAILWEAN	Requirement of a ventilator for more than 48 cumulative hours within 30 days after surgery
OPRENAFL	Renal failure requiring dialysis within 30 days after surgery
CNSCVA	Cerebral vascular accident or stroke with motor, sensory, or cognitive dysfunction for 24 or more hours within 30 days after surgery
CDARREST	Chaotic or absent cardiac rhythm requiring CPR within 30 days after surgery
CDMI	Acute myocardial infarction which occurred intraoperatively or within 30 days after surgery
OTHSESHOCK	Sepsis associated with organ and/or circulatory dysfunction within 30 days after surgery
SUPINFEC	Superficial incisional surgical site infection that occurs within 30 days after surgery
WNDINFD	Deep incision surgical site infection that occurs within 30 days after surgery
ORGSPCSSI	Organ/Space surgical site infection that occurs within 30 days after surgery
DEHIS	Wound separation that compromises integrity of closure, occurring within 30 days after surgery
OUPNEUMO	Pneumonia that occurs within 30 days after surgery
RENAINSF	Reduced kidney capacity (without requirement for dialysis) within 30 days after surgery
URNINFEC	Infection in the kidneys, ureters, bladder, or urethra, occurring within 30 days after surgery
OTHBLEED	Use of one or more units of packed or whole red blood cells intraoperatively or within 72 hours after surgery
OTHDVT	New diagnosis of blood clot or thrombus within the venous system, occurring within 30 days after surgery
OTHCDIFF	C. diff colitis within 30 days after surgery
OTHSYSEP	Sepsis within 30 days after surgery
REOPERATION1	Unplanned return to the operating room for a surgical procedure, for any reason, within 30 days after surgery

SDCTable2. Distribution of Medicaid/Uninsured Insurance Type

**Medicaid/Uninsured Insurance Total**	**3488**
County Indigent Programs, No. (%)	1200 (34.4)
Charity care, No. (%)	88 (2.5)
Medicare/Medicaid Dual Eligible, No. (%)	417 (12.0)
Medicaid, No. (%)	929 (26.6)
Self-pay <1% charges collected, No. (%)	854 (24.5)

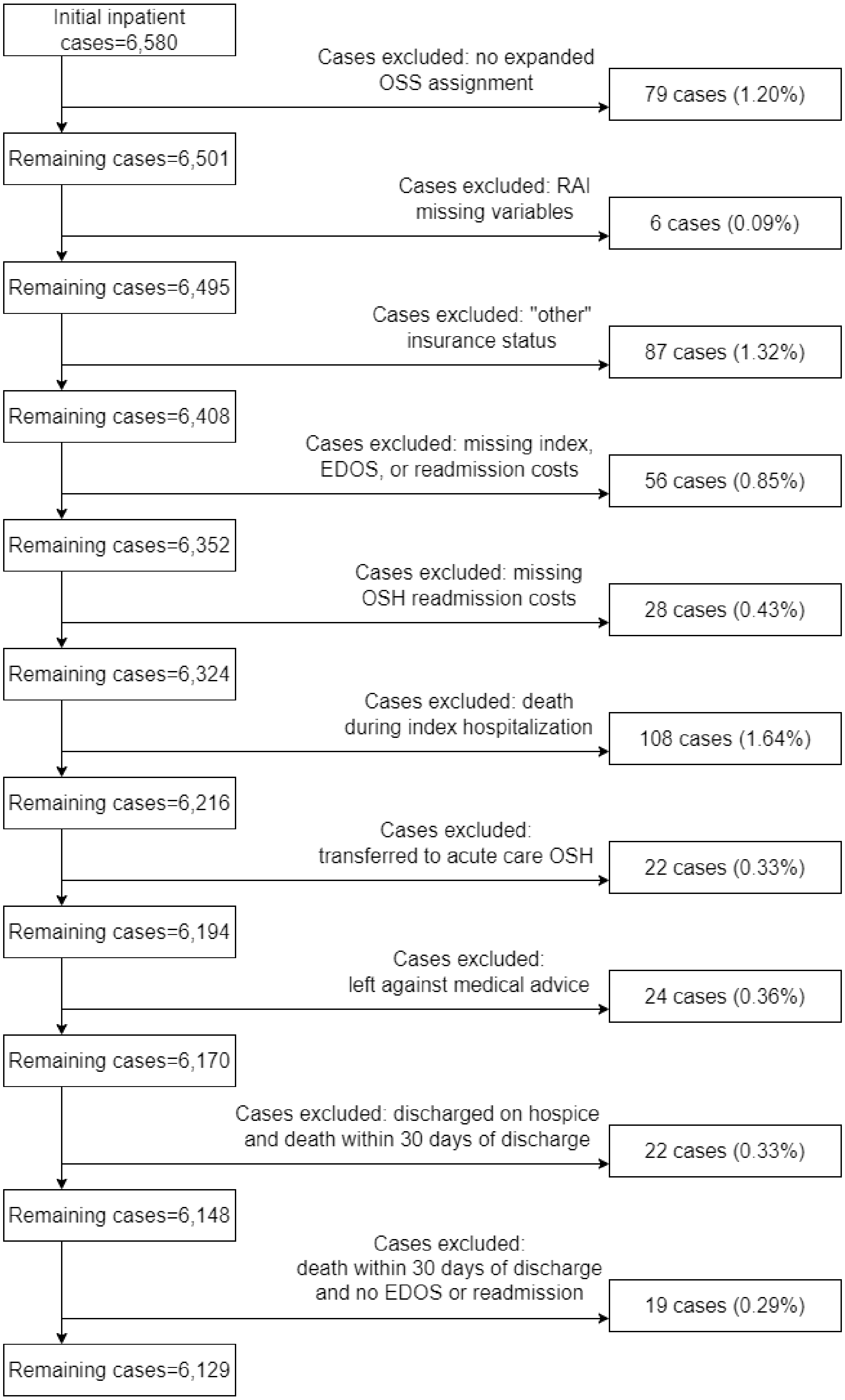

SDCFigure1. Flow Diagram of Study Cohort

National Surgery Quality Improvement Program inpatient cases from 2013-2019. Cases were excluded for having no expanded Operative Stress Score (OSS) assignment for the principal CPT code, missing variables used to calculate the Risk Analysis Index (RAI), “other” insurance status, and missing cost data. Cases lacking an expanded OSS assignment for the principal CPT code were excluded to avoid erroneously assigning a lower stress OSS based upon additional procedures that were performed. For example, a principal CPT code for a highly stressful procedure not assigned an expanded OSS could be assigned an OSS1 if the additional CPT codes contained any procedure with an expanded OSS rating. Patient mortality resulting in no or reduced chances of subsequent Emergency Department visits/Observation Stay (EDOS) and readmissions were excluded. Additional cases were excluded due to 1) death during the index hospitalization, 2) being discharged to another acute care outside hospital (OSH), 3) discharge against medical advice, 4) death within 30 days of discharge when discharged to Hospice or Home on Hospice, and 5) death within 30 days of discharge without a 30-day EDOS or readmission.

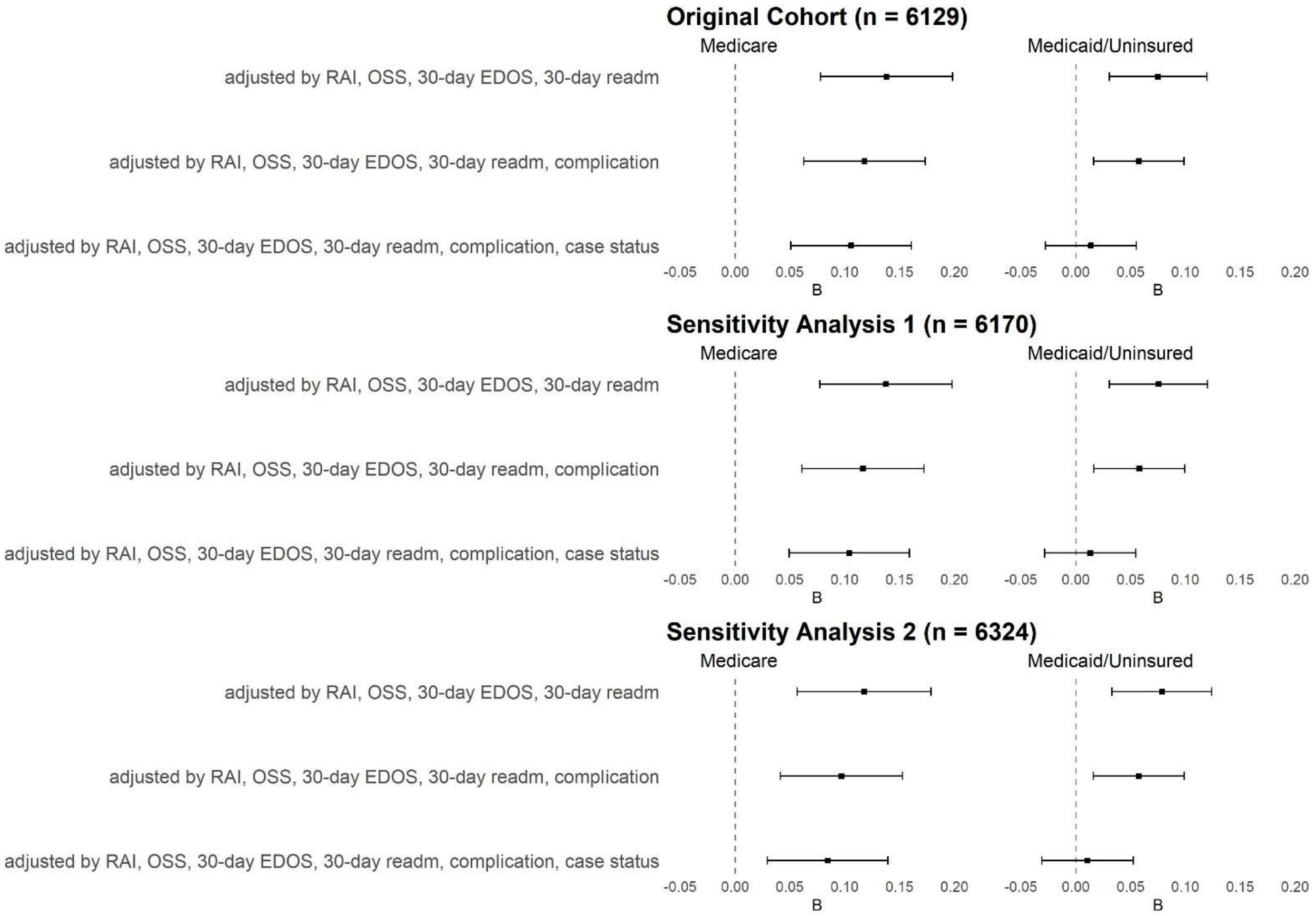

SDCFigure2. Cohort and Sensitivity Analyses of Log-Linear Regression Coefficients for Medicare and Medicaid/Uninsured Insurance Groups Compared to Private for Index Hospitalization Variable Costs

Log-Linear Regression Coefficients (B) for Medicare and Medicaid/Uninsured Insurance groups (reference Private group) for the study cohort and two sensitivity analyses. Cases were excluded from the cohort due to 1) death during the index hospitalization, 2) discharge to another acute care outside hospital, 3) discharge against medical advice, 4) death within 30 days of discharge when discharged to Hospice or Home on Hospice, and 5) death within 30 days of discharge without a 30-day EDOS or readmission. Two sensitivity analyses were performed adding exclusion groups 4 & 5 and 1-5 to the analysis to determine whether the association of insurance type was robust to cohort selection.

Abbreviations: EDOS, Emergency Department visits/Observation Stays; OSS, expanded Operative Stress Score; RAI, Risk Analysis Index; Readm, readmissions

SDCTable3. Index Hospitalization Length of Stay using 3 Nested Models (M1-M3) Adjusted for RAI, OSS, Insurance, 30-day Readmissions, 30-day EDOS, Any Complication and Case Status

	log(Length of Stay) M1				log(Length of Stay) M2				log(Length of Stay) M3			
	%change	Est	CI	p-value	%change	Est	CI	p-value	%change	Est	CI	p-value
Intercept		1.77	1.71 - 1.83	**<.001**		1.63	1.57 - 1.68	**<.001**		1.44	1.39 - 1.49	**<.001**
**RAI (Ref = Normal 21-29)**												
Robust (≤20)	-24.19	-0.28	-0.32 - -0.23	**<.001**	-20.41	-0.23	-0.27 - -0.18	**<.001**	-18.14	-0.20	-0.24 - -0.16	**<.001**
Frail (30-39)	11.99	0.11	0.04 - 0.19	**.003**	9.08	0.09	0.02 - 0.16	**.017**	8.21	0.08	0.01 - 0.15	**.021**
Very Frail (≥40)	28.03	0.25	0.10 - 0.40	**.001**	21.45	0.19	0.05 - 0.34	**.007**	11.45	0.11	-0.02 - 0.24	.110
**Expanded OSS (Ref = OSS3)**												
OSS1-2[a]	-7.74	-0.08	-0.12 - -0.04	**<.001**	-3.36	-0.03	-0.07 - 0.01	.097	-15.92	-0.17	-0.21 - -0.13	**<.001**
OSS4	48.38	0.39	0.34 - 0.45	**<.001**	31.97	0.28	0.23 - 0.33	**<.001**	38.11	0.32	0.28 - 0.37	**<.001**
OSS5	64.97	0.50	0.39 - 0.62	**<.001**	41.06	0.34	0.24 - 0.45	**<.001**	61.93	0.48	0.38 - 0.58	**<.001**
**Insurance (Ref = Private)**												
Medicare	17.29	0.16	0.10 - 0.22	**<.001**	15.23	0.14	0.09 - 0.20	**<.001**	12.07	0.11	0.06 - 0.17	**<.001**
MC/UN	28.09	0.25	0.20 - 0.29	**<.001**	26.12	0.23	0.19 - 0.27	**<.001**	14.20	0.13	0.09 - 0.17	**<.001**
**30-day Readmissions and EDOS (Ref = No Readmission or EDOS)[b]**												
Readmissions	34.68	0.30	0.25 - 0.35	**<.001**	4.03	0.04	-0.01 - 0.09	.136	4.05	0.04	-0.01 - 0.09	.111
EDOS	10.56	0.10	0.04 - 0.16	**.001**	0.96	0.01	-0.05 - 0.07	.749	1.80	0.02	-0.04 - 0.07	.526
**Any Complication**					81.24	0.59	0.55 - 0.64	**<.001**	76.43	0.57	0.53 - 0.61	**<.001**
**Urgent/Emergent (Ref = Elective)**									62.39	0.48	0.45 - 0.52	**<.001**

Abbreviations: CI, 95% Confidence Interval; EDOS, Emergency Department visits/Observations Stays; Est, Estimates; MC/UN, Medicaid/Uninsured; OSS, Operative Stress Score; RAI, Risk Analysis Index; Ref, Reference Value;

Note: %change is calculated with marginal change of Log(variable costs) for one unit of each variable change below

$(e^{(\text{intercept+estimated coefficients})} - e^{\text{intercept}})/ e^{\text{intercept}} *100$, which is equal to $(e^{\text{estimated coefficients}} -1)*100$

[a]OSS1 and OSS2 (very low and low stress surgeries) were combined due to small sample size of OSS1 procedures

OSS3 moderate stress, OSS4 high stress and OSS5 very high stress

[b]Patients with both a readmission and EDOS were assigned to the readmission group; readmissions (n=892) and EDOS (n=587)

SDCTable4. Variable Costs for 1st 30-day EDOS and 1st 30-day Readmission Adjusted for RAI, OSS, Case Status, Insurance and Any Complication

	log(1st EDOS Variable Cost)				log(1st Readmission Variable Cost)			
	%change	Estimates	CI	p-value	%change	Estimates	CI	p-value
Intercept		4.93	4.55 - 5.31	**<.001**		8.17	7.95 - 8.39	**<.001**
**RAI (Ref = Normal 21-29)**								
Robust (≤20)	-8.27	-0.09	-0.36 - 0.19	.542	-30.89	-0.37	-0.52 - -0.22	**<.001**
Frail (30-39)	46.11	0.38	-0.07 - 0.83	.100	-13.32	-0.14	-0.35 - 0.07	.186
Very Frail (≥40)	8.72	0.08	-0.90 - 1.07	.868	-18.23	-0.20	-0.56 - 0.16	.270
**Expanded OSS (Ref = OSS3)**								
OSS1-2[a]	16.19	0.15	-0.11 - 0.41	.261	7.64	0.07	-0.08 - 0.23	.349
OSS4	-6.15	-0.06	-0.37 - 0.24	.683	-10.18	-0.11	-0.26 - 0.04	.157
OSS5	119.46	0.79	0.16 - 1.42	**.015**	-2.34	-0.02	-0.33 - 0.29	.881
**Urgent/Emergent (Ref = Elective)**	-7.17	-0.07	-0.30 - 0.15	.524	20.60	0.19	0.06 - 0.32	**.004**
**Insurance (Ref = Private)**								
Medicare	0.85	0.01	-0.38 - 0.39	.966	2.22	0.02	-0.18 - 0.22	.831
MC/UN	0.47	0.00	-0.28 - 0.29	.974	5.00	0.05	-0.11 - 0.21	.549
**Any Complication**	33.54	0.29	0.06 - 0.52	**.012**	50.34	0.41	0.28 - 0.54	**<.001**

Abbreviations: CI, 95% Confidence Interval; EDOS, Emergency Department visits/Observations Stays; MC/UN, Medicaid/Uninsured; OSS, Operative Stress Score; RAI, Risk Analysis Index; Ref, Reference Value

[a]OSS1 and OSS2 (very low and low stress surgeries) were combined due to small sample size of OSS1 procedures

OSS3 moderate stress, OSS4 high stress and OSS5 very high stress

Patients with both a readmission and EDOS were assigned to the readmission group; readmissions (n=892) and EDOS (n=587)

Note: %change is calculated with marginal change of Log(variable costs) for one unit of each variable change below

$(e^{(\text{intercept+estimated coefficients})} - e^{\text{intercept}})/ e^{\text{intercept}} *100$, which is equal to $(e^{\text{estimated coefficients}} -1)*100$

SDCTable5. Mean and Median Index Hospitalization Variable Costs ($) for Elective Cases by Insurance Type Stratified by the Expanded Operative Stress Score

Number (%)*	Overall<br>2802	Private<br>909 (32.4)	Medicare<br>603 (21.5)	Medicaid/Uninsured<br>1290 (46.0)	p-value
**All Cases Mean (SD)**	10,754 (12,369)	9,962 (13,128)	12,167 (12,128)	10,651 (11,872)	**< .001**
Q1	5,162	5,001	5,660	5,201	
Median	7,373	6,723	8,645	7,277	
Q3	11,796	10,816	13,259	11,700	
**Expanded OSS1-2[a] Mean (SD)**	7,823 (8,082)	7,693 (7,067)	8,920 (10,706)	7,455 (7,405)	.622
Q1	3,903	3,696	4,056	3,954	
Median	5,614	5,522	5,927	5,574	
Q3	8,188	7,733	9,117	7,995	
**Expanded OSS3 Mean (SD)**	9,583 (10,698)	8,719 (9,487)	10,627 (11,713)	9,746 (11,008)	**< .001**
Q1	4,849	4,718	4,838	4,958	
Median	6,521	6,029	7,137	6,736	
Q3	9,924	8,691	11,081	10,185	
**Expanded OSS4 Mean (SD)**	14,567 (17,204)	13,798 (21,499)	15,698 (13,286)	14,582 (15,166)	**.015**
Q1	7,722	7,458	8,397	7,646	
Median	10,496	9,658	11,262	10,786	
Q3	14,196	12,935	15,694	14,345	
**Expanded OSS5 Mean (SD)**	16,512 (9,545)	14,541 (5,855)	17,063 (10,089)	17,517 (11,307)	.594
Q1	11,092	11,020	10,943	11,686	
Median	13,606	13,427	13,606	13,587	
Q3	17,652	15,856	19,024	17,327	

Abbreviations: OSS, Operative Stress Score; SD, standard deviation; Q1, Quartile 1; Q3, Quartile 3
Kruskal-Wallis test used for p-values due to highly skewed cost data
[a]OSS1 and OSS2 (very low and low stress surgeries) were combined due to small sample size of OSS1 procedures
OSS3 moderate stress, OSS4 high stress and OSS5 very high stress

SDCTable6. Mean and Median Index Hospitalization Variable Costs ($) for Urgent/Emergent Cases by Insurance Type Stratified by the Expanded Operative Stress Score

	**Overall**	**Private**	**Medicare**	**Medicaid/Uninsured**	**p-value**
**Number (%)***	3327	568 (17.1)	561 (16.9)	2198 (66.1)	
**All Cases Mean (SD)**	14,054 (18,759)	13,286 (19,509)	18,041 (17,946)	13,235 (18,642)	**< .001**
Q1	4,753	4,179	7,490	4,534	
Median	8,432	7,580	12,381	7,758	
Q3	15,581	13,908	22,304	14,474	
**Expanded OSS1-2[a] Mean (SD)**	10,453 (13,981)	9,744 (15,031)	14,030 (17,240)	9,848 (12,810)	**< .001**
Q1	3,612	3,076	5,355	3,562	
Median	6,155	4,751	10,127	5,749	
Q3	11,864	10,078	17,657	11,203	
**Expanded OSS3 Mean (SD)**	14,589 (20,406)	13,749 (20,626)	19,096 (18,930)	13,572 (20,589)	**< .001**
Q1	5,165	4,595	7,685	4,922	
Median	8,496	7,466	12,671	7,944	
Q3	15,061	13,586	22,275	13,917	
**Expanded OSS4 Mean (SD)**	21,854 (21,082)	19,859 (23,599)	23,434 (14,535)	21,993 (21,870)	**.003**
Q1	10,051	9,116	12,798	9,747	
Median	15,083	13,903	19,602	15,017	
Q3	24,552	19,226	29,515	24,029	
**Expanded OSS5 Mean (SD)**	27,776 (24,521)	22,230 (16,947)	22,675 (8,770)	34,474 (33,656)	.482
Q1	13,488	11,345	16,680	13,717	
Median	19,707	15,981	21,535	19,765	
Q3	13,488	25,193	26,501	38,838	

Abbreviations: OSS, Operative Stress Score; SD, standard deviation; Q1, Quartile 1; Q3, Quartile 3
Kruskal-Wallis test used for p-values due to highly skewed cost data
[a]OSS1 and OSS2 (very low and low stress surgeries) were combined due to small sample size of OSS1 procedures
OSS3 moderate stress, OSS4 high stress and OSS5 very high stress
